# Forced Friends: Why the Free Energy Principle Is Not the New Hamilton’s Principle

**DOI:** 10.3390/e26090797

**Published:** 2024-09-18

**Authors:** Bartosz Michał Radomski, Krzysztof Dołęga

**Affiliations:** 1 Institute for Philosophy II, Ruhr-Universität Bochum, D-44780 Bochum, Germany; 2 Center for Research in Cognition & Neurosciences, Université libre de Bruxelles, B-1050 Brussels, Belgium; krzysztof.dolega@ulb.be

**Keywords:** free energy principle, Hamilton’s principle, similarity, analogy, formal analogy, equivalence, philosophy of science

## Abstract

The claim that the free energy principle is somehow related to Hamilton’s principle in statistical mechanics is ubiquitous throughout the subject literature. However, the exact nature of this relationship remains unclear. According to some sources, the free energy principle is merely similar to Hamilton’s principle of stationary action; others claim that it is either analogous or equivalent to it, while yet another part of the literature espouses the claim that it is a version of Hamilton’s principle. In this article, we aim to clarify the nature of the relationship between the two principles by investigating the two most likely interpretations of the claims that can be found in the subject literature. According to the strong interpretation, the two principles are equivalent and apply to the same subset of physical phenomena; according to the weak interpretation, the two principles are merely analogous to each other by virtue of their similar formal structures. As we show, adopting the stronger reading would lead to a dilemma that is untenable for the proponents of the free energy principle, thus supporting the adoption of the weaker reading for the relationship between the two constructs.

## 1. Introduction

The free energy principle (FEP for short) has recently gained a significant following across multiple disciplines ranging from neuroscience and psychology to philosophy and even biology. The principle postulates that all living organisms belong to a special subset of self-organizing systems that ensure adaptive exchanges with the environment by maintaining their own states within bounds prescribed by their phenotype. This is usually modeled as the minimization of divergence between the organisms’ expected and actual states, which is formally equivalent to the minimization of the long-term average of an information-theoretic quantity of expected free energy.

Although the FEP and the landscape of related literature have changed over the years, one of the leitmotifs of the scientific discourse around the principle are the claims about its similarity to Hamilton’s principle (or HP for brevity) in statistical mechanics. For example, some publications state that, under the FEP, the brain [1] or even whole living systems [2] (Appednix A, Box 2) conform to Hamilton’s principle. Recent publications assume that the FEP’s link with HP is so strong that it vindicates renaming the free energy research project to ‘Bayesian Mechanics’ [3,4,5].

However, despite the pervasive nature of the claims about the FEP’s connection to HP, little attention has been given to spelling out the exact nature of the relationship linking the two principles. In this article, we aim to fill this gap in the literature and investigate two possible relationships that might link the FEP to HP. As we will show, the purported similarity between the two constructs is quite limited. Those trying to argue for a deeper connection face an untenable dilemma: Either the FEP is equivalent to HP (a system conforms to the free energy principle if and only if it conforms to Hamilton’s principle) and does not apply to biological systems or it applies to biological systems but is not logically equivalent to HP. Against this dilemma, we propose a weaker relationship of formal analogy. While this interpretation fails to deliver many of the payoffs promised by the stronger interpretation, it is much closer to what (at least some) free energy theorists seem to be after.

To achieve this goal, we begin with an overview of Hamilton’s principle in Section 2 and the reasons that may motivate researchers to link their work back to this principle. This is followed by a brief exposition of the FEP’s core claims in Section 3.1. The rest of Section 3 provides a more detailed discussion of the two directions in which comparisons between the FEP and HP can be made: one moving from a formal process that is compliant with HP and applying it to living organisms (Section 3.2), another moving from a biological process and re-describing it in a way that is supposed to be compliant with HP (Section 3.3). Having discussed how the connection between the two principles has been established in the literature, we pose a simple question: What sort of connection is it exactly? In Section 4, we suggest two possible interpretations consistent with FEP literature: a strong reading of logical equivalence (Section 4.1) and a weak reading of analogy (Section 4.2). In subsection “Dilemma for the Strong Reading”, we point out that an equivalence relation leads to a two-horned dilemma, in which proponents of this interpretation must pick one of two unwelcome alternatives. On the first horn, if the FEP is logically equivalent to HP, then it does not apply to real (non-conservative) biological systems. On the other horn, if the FEP does apply to such systems, then it cannot be logically equivalent to HP. Having presented this dilemma, we then consider an alternative interpretation, according to which the FEP is analogous to HP. In subsection “Assessing the Analogies between the Free Energy Principle and Hamilton’s Principle”, we show that the relationship of analogy might not only provide a good description of the formal similarities between the two principles but also match recent claims about their relation. This interpretation, however, does not support stronger claims about the epistemic role or status of the FEP within cognitive science. We conclude by summarizing our main claim and pointing to some outstanding issues that could not be addressed in the current paper.

## 2. Hamilton’s Principle of Stationary Action

Hamilton’s principle is a formal construct in classical mechanics [6,7,8]. In this field, every law of motion can be represented by a set of differential equations that describe the relations between changes or differences in specified quantities [9]. These laws are often first formulated in the integral form, e.g., *∫ f*(*x*) *dx*, because empirical observations involve finite systems that delimit an integer. They may be later generalized to a differential form, e.g., *d/dx f*(*x*), which allows for wider applicability. To reformulate integral laws into differential laws, one can use variational principles. Conveniently, nearly all such laws of motion can be obtained from one principle [9,10].

**Figure 1:**
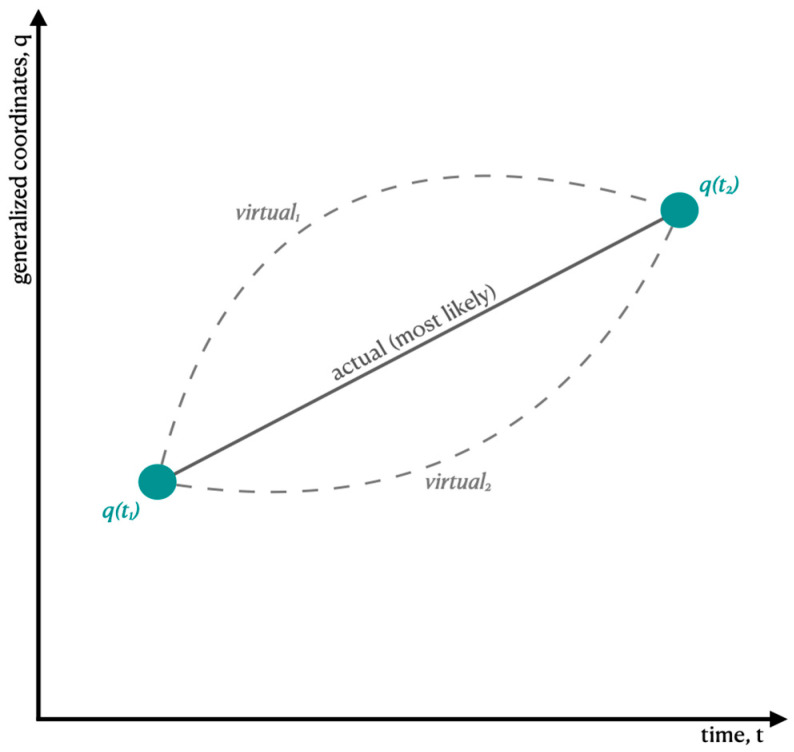
Variations of paths under HP. By knowing the start and end points as well as the kinetic and potential energies of a particle traveling from point *q*(*t*_1_) to *q*(*t*_2_), we can compute possible paths that the particle may take by varying positions and momenta of the particle. The most likely path, where action is stationary, is denoted as the actual path. Here, being stationary means the derivative of the action is zero.

HP states that the motion of a physical system can be determined by finding an *extremum* of a functional of the *Lagrangian*. ‘Extremum’ means either a minimum or a maximum, and ‘functional’ means a function of a function, e.g., *f*(*g(x*)). A Lagrangian is a function that specifies the physical system’s position coordinates and their rates of change [11]. Typically, the function of position is identified with the system’s potential energy, and the function of velocity is identified with its kinetic energy. Thus, in classical mechanics, the Lagrangian is equal to the difference between potential energy and kinetic energy which, when integrated over time, defines *action*. HP applied to classical mechanics usually yields the prediction that the most likely path for a system to take is the one where the action is minimized, which is why it is commonly referred to as the ‘least action principle’ (see Figure 1 for a helpful illustration of these ideas).

At the most fundamental level of physics, where all physical processes are conservative, HP can be applied universally [8]. The all-encompassing scope of HP led some to view it as fundamental, both ontologically—as a statement about the most basic ‘nature’ of the universe [10,12]—and epistemologically—as a conceptual foundation of virtually all models in physics [7]. However, this reverence for HP, and variational principles more generally, also stems from scientists perceiving them as being ‘aesthetically and mathematically very appealing‘ [10] (p. 1). Their formal elegance serves to simplify and facilitate mathematical operations, proofs, and model development [7] (p. 12) and allows for a more intuitive and natural physical interpretation of the mathematical variables [13].

Having said that, conservative principles like Hamilton’s do not work for biological systems (here understood as living organisms). Organismal behavior is characterized by non-conservative and time-irreversible processes [14]. These characteristics are the cornerstone of the ‘modern‘ view of biological systems, which is dominated by instability, fluctuations, and evolutionary processes [15]. According to this portrayal, systems may evolve towards increased complexity as a result of non-conservative and time-irreversible processes. In order to account for non-conservative systems (e.g., organisms), a dissipation function must be specified. Intuitively, if a system loses energy to its environment, then we need to specify the rate at which the loss occurs. In this way, by using ad hoc techniques, some extensions of HP are possible in selected special cases [16]. However, because of the non-linear regime of such transformations, no general method, or indeed methodology, exists for obtaining dissipative evolution equations [17]. As a result, HP, which assumes conservation of energy and full-time-symmetry of target systems, does not have biological phenomena in its scope of application. In other words, Hamilton’s principle applies only to conservative systems. By “conservative”, we mean systems whose total energy remains constant over time.

## 3. The Free Energy Principle as a Principle of Least Action

We have counted at least ten instances of academic publications where the FEP and HP are compared, though this is likely an underestimation [2,18,19,20,21,22,23,24,25,26,27]. Not only are these claims varied, but they also take the form of brief and sometimes ambiguous remarks rather than full-blown arguments. For example, Friston, in his 2008 article, states that the optimization scheme following from the FEP ‘[…] is basically a restatement of Hamilton’s principle of stationary action’ (p. 22). Ramstead and colleagues, on the other hand, write that ‘the FEP is a non-substantive principle that can be applied to any biological system in general (much like Hamilton’s principle of least action)’ [28] (p. 192). Finally, Parr et al. claim that the FEP is simply ’the Hamiltonian principle of least Action applied to behavior.’ [27] (p. 49).

The diversity of claims about the FEP’s relationship to HP makes selecting a target for a comprehensive critique extremely difficult, especially if one wants to avoid making a straw-man argument against the proponents of the FEP. This is why, instead of focusing on just one claim, we decided to provide examples of two general strategies used for establishing a connection between the FEP and HP. Each of these strategies presents different ’directions-of-fit‘ between the two principles. In the first case, discussed in Section 3.2, a formal operation entailed by the FEP is re-described as a process that can be understood as a version of HP. In the second case, presented in Section 3.3, a biological process that is assumed to be within the scope of the FEP is formally re-described in a way that is supposed to be compatible with HP. However, before we move on to the illustrations of how the FEP is applied in research, we will first describe what we understand to be the two main claims that make up the FEP.

### 3.1. The Free Energy Principle

Although the exact mathematical form of the FEP has evolved over the years (compare, e.g., [18,29]), variational calculus has remained at the formal core of the different guises of the principle. The aim of this section is to spell out two aspects of the FEP that have been identified as its primary claims or prescriptions and are common to all of its applications. For the purpose of this brief overview, we will follow the formulation offered by Mann, Pain, and Kirchhoff in their recent overview of the FEP [30]. However, we would also recommend consulting other works [31,32,33] for more elaborate and technical expositions of the principle.

The first and perhaps the oldest claim about the FEP is that it specifies the general principle by which the brain approximates Bayesian inference [29] through the minimization of an informational quantity known as variational free energy. This quantity, following [30] (p. 6), can be defined as:

(1) $$F\left(p,\;q,\;x\right)=\sum_{w}q(w)log\frac{q(w)}{p\left(w\right)}+\sum_{w}q\left(w\right)log\frac{1}{p(x|w)}\;$$

where *w* is the hidden variable, typically interpreted as a state of the world external to the system being modeled; *x* denotes a known random variable, typically interpreted as a perceptual state of the system; *p* denotes probability distributions that make up the generative model, i.e., distributions over *w* and *x*; and *q* is the variational (i.e., approximate posterior) density over *w* parametrized by the observed states and usually interpreted as the system’s belief about the world (where ‘belief’ is used in a deflationary sense).

Formula (1) defines variational free energy *F* as a sum of two complex terms. The first addend is a measure of relative entropy between the variational density *q*(*w*) and the probability distribution *p*(*w*), which can also be expressed by means of the Kullback–Leibler divergence [24] (p. 20) and acts as a penalty for overfitting the model to the current observation [30] (p. 33). The second addend is a measure of (or rather the inverse of) the accuracy of the model and therefore can be understood as a penalty for failing to explain current observations. Under this formulation, the general aim of any system performing inference is to find a distribution *q(w)* such that the sum of the terms defined in (1) is kept as low as possible, thus minimizing *F*.

As Mann, Pain, and Kirchhoff point out [30] (p. 33), the application of the FEP to *perceptual* inference can be summarized in a simple prescription:

(2) $$q(w)\leftarrow \underset{q}{argmin}\;F.$$

One way of interpreting this prescription is that, given model parameters, the lowest possible value of *F* designates the ‘trajectory’ through the parameter space in which the tradeoff between the two constraints (model complexity and accuracy) is optimal.

At this point, it is important to acknowledge that the formulation of the FEP in (1) is simply a restatement of the Bayesian method of variational inference, where the negation of *F* is used as the evidence lower bound or ELBO [32]. As such, it is commonly interpreted as prescribing a form of model optimization that can be used to model perceptual processing in neural networks [34]. Crucially, applications of the above formulas are limited to problems of inference that do not assume any form of reciprocal influence or feedback relationship between the states of the observed variables and the states of the hidden variables. In other words, (1) and (2) only apply to problems that have been previously labeled as *passive inference*, i.e., cases in which the modeled system cannot act on or re-sample the available sensory data but merely update its beliefs about the hidden variables.

The second claim and perhaps *the* main innovation brought about by the FEP is the application of the same kind of inference to action, thus giving rise to what is commonly referred to as *active inference* [24]. This innovation is based on a simple observation that, while perception is aimed at inferring the present state of the world, agential action is aimed at modifying the state of the world. Formally, this kind of inference takes a very similar form to the variational inference described by (1), except that the quantity that is being calculated is not free energy as such but the *expected free energy G* parametrized on actions *z*:

(3) $$G\left(p,\;q,\;z\right)=\sum_{w}q\left(w|z\right)log\frac{q\left(w|z\right)}{p\left(w\right)}+\sum_{w}q\left(w|z\right)\sum_{x}p\left(x|w\right)log\frac{1}{p(x|w)}.$$

Just like in the case of (1), expected free energy is a sum of two terms. However, here, the first addend is interpreted not as a discrepancy between the model and the world but rather as a measure of divergence between the state of the world and what is now seen as system’s preferences over states of the world given action policies (*q*(*w|z*)). The second addend in (3) is a term representing the failure to minimize the surprise of *x*, which here is interpreted as a future sensory state (under the assumption that states of the world are causally responsible for the sensory states). As in the case of mere inference, the FEP applied to action results in a simple principle [27]:

(4) $$z\leftarrow \underset{z}{argmin}\;G.$$

There are two important points of note related to (3) and (4). First, although the general formulation and prescriptions made by the FEP can be summarized by Equations (1)–(4), the latter two equations subsume the former ones since minimizing *G* requires the minimization of the surprise of future sensory states, thus ensuring that *F* will also be minimized. Second, the shift from inferences over *mere* observations to ones involving preferences or policies for actions is what warrants treating model systems as *agents*, though the definition of agency employed by the FEP is rather minimal. In the active inference modeling framework, an agent is simply a system that emits actions; it becomes an active inference agent if it has a model of its environment (what we have previously referred to as ‘beliefs’) and can control its action–perception loops to bring about the expected (and, simultaneously, preferred) sensory observations [27] (pp. 3–6). From this perspective, the FEP offers a modeling approach that is similar to reinforcement learning, albeit with expected free energy taking the place of reward and cost functions [35] (with some authors claiming that it can even explain hedonic valence [36]). Although, the application of the term ‘active inference agent’ is intended to cover any living organism from bacteria to humans [27] (p. 7), from a mathematical point of view, such an agent is identified merely as a weakly mixing random dynamical system with an attracting set [37] (p. 4) (we return to this issue later).

Since the formulation of the above claims, much work within the FEP community has been spent on delineating agents from their environments (mostly with the use of Markov blankets) and adding auxiliary assumptions aimed at spelling out the conditions under which FEP-compliant agents can exist (see [27,37] for a definitive overview), though many of these assumptions have been argued to be too constrictive for modeling living organisms [32,38]. However, in order not to get bogged down by the details of these changes, we can sum up this section in the following way: Under the FEP, all (real) biological systems are modeled as (abstract) dynamical systems, albeit with certain specific properties (weakly mixing or ergodic, self-organizing, reaching a non-equilibrium steady state) and with an arbitrary, model-specific state space. In active inference, a weakly mixing system is defined as a system that converges towards an invariant set of states (an attractor), also called a non-equilibrium steady state, where the average of surprisal over time is at its minimum. The process of reaching the invariant set/attractor/steady state is usually described as the process of self-organization [34] (p. 498) and involves minimizing the quantity of the expected free energy [35,36].

### 3.2. Model Inversion

Model inversion is one of the first notions through which a connection between the FEP and HP has been made [19,23]. Model inversion is a notion from statistics and machine learning. It is commonly defined as the task of learning a mapping from some set of observed states to model parameter distributions that approximate the posterior distribution over the hidden states responsible for the observations [39].

In the FEP literature, model inversion is first given a biological interpretation, then described in information-theoretic terms, and finally interpreted as a problem of finding the most likely trajectory of motion akin to problems in classical mechanics. The FEP literature thus draws on a long tradition in psychology and neuroscience, which poses model inversion as the problem that brains need to solve (see [40] for a closer discussion of the historical background). However, making the connection between model inversion and the brain requires an additional step. The latter interpretation frames model inversion as an inference problem. Here, the goal is to infer the state of a latent variable, usually interpreted as the hidden cause of some observable state. This estimation is based on a prior model of the possible relations between the hidden and the observable states. Predictably, the solution to this problem is usually specified in terms of variational approximation of Bayesian inference, which turns an intractable problem of exact inference into an ‘easy optimization problem’ [18] (p. 7).

In order to justify a biological application, Friston [19] (p. 2) presents a general scheme for data modeling that may be used to subsume many different models of data of arbitrary complexity under a single hierarchical model. Then, he shows that every model of this kind can be inverted using the same optimization scheme. Finally, he argues that such a form of model inversion could be implemented in a neural substrate, which should encourage us to view the structure and function of the brain as an inference machine [19]. This last move is motivated by drawing on earlier neuroscientific work [41] and postulating that predictive coding schemes could serve as a plausible neural implementation of generic variational schemes for the approximation of Bayesian inference. The generic variational scheme for inversion itself is labeled as ’basically a restatement of Hamilton’s principle of stationary action‘ [19] (p. 22). This is further elaborated in Friston and Kiebel’s 2009 [23] article, where the process of learning is formalized as a gradient ascent that conforms to HP. Significantly, in both cases, it is the mathematical approximation scheme (which recasts the inference problem as an optimization one) and not the actual functioning of the brain (supposedly trying to optimize variational free energy) that is claimed to be a restatement of HP. However, these initial claims about formal similarity eventually evolved into the claim that all biological systems, in order to resist dispersion, must minimize the long-term average of (expected) free energy, which is interpreted as the same quantity (action) that is minimized in HP. This is expressed clearly in passages such as: ‘[L]ong-term average of free-energy […] optimized by evolution, development and learning […] is called action in physics. This means the free-energy principle is just an example of Hamilton’s principle of least action‘ [1] (p. 221), or ’living systems conform to Hamilton’s principle of least action via active inference‘ [2] (Appendix A, Box 2).

The formal aspect of the comparison that allows for drawing similarities between the FEP and HP is model inversion. Inverting the model can be seen as a kind of motion through a ‘hypothesis space’, where points in the space represent hypothesized mappings between latent variables and sensory outcomes, and the path through that space ‘traces’ which hypotheses have been explored. Thus, thanks to a few assumptions about the model and its variables (such as the known and unchanging nature of the model, no cost to learning, and the type of distributions that are to be learned), the problem of model inversion can be restated in a way that resembles a problem of finding a path of stationary action. More specifically, model inversion can be interpreted as a problem of finding a path that runs along the true means of the distribution over hidden causes. In this setting, the (inversion) problem of learning the posterior is expressed as the problem of finding a trajectory of states (parameters) that minimizes the gap between the estimated (known) posterior and the actual (hidden) posterior. The solution to this learning problem is to find the path of the approximate posterior, which runs exactly along the trajectory of the true conditional means. Conveniently, such a solution can be found by selecting the path on which the integral of free energy over time is at its minimum. Thus, the problem’s set-up and its solution clearly resemble those prescribed by HP (as has been stated by Friston in the 2008 passage quoted at the beginning of Section 3).

To sum up, we began with a process known from statistical inference and machine learning—model inversion—and have then recast it as a problem of approximate Bayesian inference. This reinterpretation suggests model inversion as a plausible description of the task of perceptual learning, allowing us to view the brain as an inference machine. At the same time, the problem of inverting the model is presented as a problem of finding an optimal trajectory. This variational approximation scheme for model inversion is argued to be a restatement of HP. Thus, in the case of model inversion, the connection between the FEP and HP can be seen at two levels: at the exposition level of the target phenomenon, where learning is re-described as motion through space (a canonical problem for Hamiltonian mechanics) and at the formal level of modeling, where optimization is performed in accordance with variational principles. The problem description and the methods used to solve it speak to a close relation between the principles at an abstract level. However, nothing about model inversion or its restatement answers the question whether the brain literally performs model inversion in a way that conforms with the FEP (and by extension with HP via formal equivalence). As such, the issue of the two principles’ applicability to living systems remains unresolved, even though we are told to believe that if biological systems are found to conform to the FEP, then they will actually conform to HP as well.

### 3.3. Morphogenesis

Morphogenesis—a distinctly biological phenomenon of shape formation in a tissue or an organ [42]—is the second notion through which a connection between the FEP and HP is made. In the example we discuss [43], the FEP is portrayed as an overarching concept and a building block of a novel ‘first principle’ approach for predicting morphogenetic ‘movements’.

Compared to the previous example, the reasoning behind introducing free energy models of morphogenesis is inverted: First, morphogenesis is argued to be adequately subsumed under a version of HP; then, it is re-described as cellular decision-making, which in turn can be simulated in silico as a form of free energy minimization. This move is argued to license the use of a variational approach to generate predictions for morphogenesis. In what follows, we demonstrate how, starting from a biological phenomenon and giving it a free energy interpretation, some authors attempt to secure the relation between the FEP and HP.

The first step is to argue that there exists a variational principle, to which the cells undergoing morphogenesis comply. Kuchling et al. [43] assume that ‘the least action principle can predict the emergence of form’ by prescribing an optimal, i.e., ‘least action’, trajectory of shape formation in a biological system (p. 93). However, the principle in question is not Hamilton’s principle but rather a version of it, one which is supplemented with a Rayleigh dissipation function. Rayleigh’s function applies only in cases where the dissipative force is local in time, is linearly dependent on velocity, and the velocities themselves are low [44]. This small addition changes the scope of the principle of least action from computing paths of individual particles to finding an average motion of an ensemble of particles [43] (pp. 93–94). Thus, if a biological system is interpreted as a collective of Rayleigh-dissipative systems, then it may on average follow the path of least action and comply with a version of HP.

Next, in order to transition from morphogenesis as a process following a path of least action to a process minimizing variational free energy, the crucial step is the reformulation of action as the path integral of the marginal likelihood or self-information. Thanks to this maneuver, ‘the principle of least action manifests as a principle of least internal entropy‘ [43] (p. 94). This equivalence allows the Lagrangian (a full description of motion) of the ensemble to be replaced with a variational free energy functional [43] (pp. 95–96). As a result, Kuchling et al. argue that ‘the variational free energy that is being minimized in Bayesian inference follows out of classical analytical and statistical physics considerations as a unique form of a least action principle’ (p. 104) and hence that ‘the variational free energy minimization in active inference is related to the variational principle of least action’ (p. 105). However, this claim only refers to the formal equivalence of the FEP and HP under a rather demanding assumption that the action minimized in HP is identical to the surprise minimized in active inference.

In active inference, the modeling problem of exact inference, which involves intractable computations of surprise, is replaced by a more manageable optimization problem of minimizing expected free energy that effectively becomes a cap or an upper bound on surprise. Similarly, here, it is a computationally challenging problem to model an open and dissipative system as directly minimizing action. Instead, it is argued that the dynamics of such a system can be computed by minimizing the upper bound on action, which, by our definition, is the expected free energy. As a consequence, morphogenesis is described as an inference process [43] (p. 104) using ‘top-down’ or ‘goal-seeking’ models that obey the ‘optimality principle’ of variational free energy minimization (but see also [45]).

And so, the mathematical reinterpretation begins with a translation of the real system’s characteristics into a mathematical object in the model. A real cell is described as a system in a certain state configuration or position in an abstract space. Consequently, its biochemical activity is described as a change in the system’s state configuration. Since each configuration occupies a distinct position in the arbitrary space, the real cellular processes can be viewed as motion through the state space. Finally, the dynamics of this movement are defined in terms of equations of motion—differential stochastic equations [43] (p. 89).

Thanks to the mathematical re-description, the biochemical changes can be associated with probabilistic, information-theoretic terms. And so, each physical state turns into a parameter (a mean) of probability densities, the biochemical activity comes to be understood as a change in the encoding of the densities, and action becomes synonymous with the gradient flow towards a minimum of an information-theoretic expected free energy [43] (p. 89). This means that, much like when constructing a variational principle, the modeler can choose the parameters, the set of possible configurations of the parameters, their motions, as well as the general equation governing this motion, which in this case is taken to be identical to free energy minimization. Consequently, the state space is a property of a model entirely determined and disclosed by the modeler.

The FEP, viewed solely as a statement about the mathematical properties of weakly mixing systems, does not constrain the set of variables and their possible configurations (the state space) used to describe these systems. In such a form, the FEP also does not impose any semantic interpretation for these variables. It is merely a claim that in a transition between arbitrary configurations, the actual path is where the energy functional is stationary, that is, where the expected free energy is at minimum. The interpretation of the mathematical variables, their configurations, and transitions is not predetermined but varies in each individual model.

What is crucial for the current discussion is that the resulting mathematical analysis is not a description of the real phenomenon itself. In a modeling framework, the system of focus is essentially a mathematical object. For such an object, certain propositions are specified as true by the modeler. In our example, morphogenesis is re-described in the Bayesian framework as a process of cellular decision-making. The idealizing assumptions about a mathematical object and its linearly dissipative dynamics in the FEP model are attempts at reflecting the open and non-conservative nature of real biological cells. Let us explicitly restate these assumptions and discuss their implications for the relationship between the FEP and Hamilton’s principle.

The first assumption made by Kuchling et al. is the addition of dissipation. The Rayleigh dissipation function that Kuchling and colleagues use is a popular assumption, albeit one with limited applicability. It accounts for friction [46] (p. 12), so a non-conservative force that is assumed to be proportional to velocity. Rayleigh himself intended for his function to ‘approximate a law’ of friction in vibration (periodic motion) but was aware that his function could not be trivially extended to cases of dissipation through heat [47]. This issue is still recognized in contemporary physics, where the function is considered as not ‘sufficiently comprehensive’ to ‘describe systems with more general dissipative features like history dependence, non-locality, and non-linearity that can arise in open systems’ [48] (p. 1). Therefore, Rayleigh dissipation may be more appropriately seen as an approximation [11] or even a way to formally imitate a non-dissipative mathematical structure [8], rather than a biologically plausible characterization of a feature found in living systems. 

The second assumption adopted by Kuchling et al. is to consider action as a description of an ensemble of particle systems. Individually, particles do not comply with the modified principle of least action because they are subject to non-linear dissipative forces, which act as constraints on the particles’ behavior. Instead, the motion is averaged of several particles, so that the whole ensemble becomes the new system of analysis. Thus, the focus is placed on describing the ensemble of particles as tending (on average) towards least action. Noisy constraints are abstracted and averaged out.

This modeling distortion is arguably legitimate because, over long periods of time, the larger system of interest will follow the least action path. However, the ‘long period’ in question may actually mean ‘billions of years or indefinitely’ [49] (p. 373). This is yet a further problem for Kuchling and colleagues’ proposal. When an open system is considered, its path towards the minimum of action may not be plausibly verifiable. Instead, such a system will be in a process of constantly acquiring and subsequently dissipating as much energy as possible on the path toward the action minimum. Whether or not such a system will tend towards minimizing action is unknown to us, either because the minimization would take eons or because there could be some dissipative forces (constraints) that prevent action minimization in the time period relevant for modeling the target phenomenon. In short, the assumption made by Kuchling et al. is that an open system over a long time-span would generally direct the flow of energy towards minimizing action. Still, this claim remains an ad hoc hypothesis. Even if we were to assume it to be true, it could not be realistically verified.

Even if we put such epistemological difficulties aside, the shift from individuals to ensembles changes the definition of what constitutes a system and which constraints should be taken into account. This makes it easy for the authors to set up their model in a way that trivially satisfies the ‘least action’ requirement. For example, if a system we want to study non-linearly loses energy to another system, then we can just try to describe them as parts of a larger system that on the whole has linear dynamics. If that larger system also turns out to be non-linearly dissipative, then we may redraw the system boundaries yet again to include more parts. As a result, the main feature of Kuchling et al.‘s approach (and the crux of the problem) is that it is based on a reinterpretation of what the system minimizing action is.

The ensemble assumption comes with the cost of obscuring the real object that is being targeted by the model. In the case of morphogenesis, we began at the level of a cell. However, for the sake of modeling, we are told that individual cells do not matter in the grand scheme of things. It is only their ensemble, on average, that will behave as a collective free energy minimizer. However, without an appropriate theory to specify which groupings of cells are legitimate and which are not, the formal descriptions of the mathematical object in the model become largely arbitrary.

As we mentioned at the beginning, the transition from describing morphogenesis as the least action process to treating it as a free energy minimizing one is allowed by substituting the Lagrangian for the variational free energy functional. For this to work, Kuchling et al. must presuppose that ensembles of cells are a special case of an open dissipative system for which a Lagrangian function exists. This, in turn, entails that the action functional exists and that the modeled (target) system will conform to HP. The existence of a Lagrangian is a non-trivial assumption and, as such, requires justification or at least an explanation of how it relates back to real systems (i.e., cells, organs, organisms, and so on).

Furthermore, the mathematical object is argued to be weakly mixing, and hence ergodic, meaning that over an infinite time horizon, it will eventually explore every possible state in its state space. Additionally, the ergodicity of the system means that the time spent in each region will be proportional to the probability of finding the system occupying that region of space at any point in time (e.g., 30% of time spent in region A will correspond to a 30% chance of finding the system in that region at any time). The universal applicability of the ergodic assumption is still questioned, and it is not known which real-world phenomena are ergodic or whether such phenomena can be known to be ergodic at all [50,51]. Critically, however, systems that are dissipative have not been proven to be ergodic [52], underscoring the idealizing nature of Kuchling et al.’s assumptions.

Finally, a closer reading of Kuchling et al.‘s paper [43] reveals that the original bold restatement of morphogenesis as Bayesian inference amounts to a model that is rather modest in terms of its scope. The model can simulate two effects that were obtained in vivo in the manipulation of morphogenesis. In this context, the FEP is said to provide ‘an essential quantitative formalism’ [43] (p. 88) that can simulate the results of experimental interventions. In other words, we are offered a computer simulation based on the FEP that can be related back to the biological phenomenon, but only if we are willing to adopt a particular view of morphogenesis as a form of Bayesian inference. However, the modeling assumptions about the type of dissipation present in cells and the least action tendencies of cellular ensembles make it unclear how Bayesian inference could provide a biologically plausible account of morphogenesis in the first place. Taken together, this provides grounds for doubting whether the close relationship between the FEP and HP is empirically relevant for studying and predicting morphogenetic processes.

## 4. Interpreting the Relationship between the Free Energy Principle and Hamilton’s Principle

In the previous section, we have shown that, depending on the research questions and the aims of a scientific model, the connection between the FEP and HP can be presented using different directions of fit. In this section, we will focus on a general question underpinning these two ways of framing the connection between the two principles, namely: What exactly is the nature of the relationship between the FEP and HP?

In Section 2, we have indicated that it may be desirable to liken a new scientific construct, such as the FEP, to HP due to its standing in physics. In the following section, we have shown that even though the FEP literature offers plenty of claims about the connection or similarity between the two principles, there seems to be no single received view about the basis for such comparisons (Section 3). Mindful of this problem, we suggest two provisional readings of the relation between the principles: that of a strong logical equivalence (Section 4.1) and a weaker, analogical relation (Section 4.2), which seem to best fit claims that can be found in the literature. According to the former, strong reading, whenever HP holds, the FEP holds, and vice versa. According to the latter, weaker reading, the FEP may be formally and methodologically analogous to variational methods in physics that apply only to conservative systems characterized by local, well-defined measures (e.g., the temperature of an ideal gas undergoing a small change in pressure). An example of such a method is the minimum entropy production principle that approximately describes dissipative structures near balance when the energy loss is linearly related to the magnitude of the energy flow (i.e., if dissipation is linear) [53]. Thus, we suggest viewing the FEP as a claim that is analogous to HP but holds only locally under a set of idealizing assumptions and is not valid globally [38]. We will now present the pros and cons of each of these interpretations.

### 4.1. Strong Reading

Equivalence is the first and perhaps the most intuitive way of understanding statements about the FEP being ‘a version of’ [27] (p. 61) or ‘equivalent to’ HP [2]. Within mathematics and classical logic, the relation of *logical* equivalence has traditionally been understood as obtaining between two propositions if, and only if, they have the same truth value *in every* model (here understood in terms of set-theoretic structures; see [54] for the full background). Thus, logical equivalence goes beyond mere *material* equivalence (a biconditional relation between two propositions) because it is based on a formal connection which states that both relata will have the same truth value as a matter of necessity. In the case of physical principles, like the FEP and HP, such an equivalence relation may be intuitively understood as a perfect overlap of the set of models that comply with the FEP and the set of models that comply with HP. In other words, if the two principles are indeed equivalent, then for any model to which one of the principles is applicable the other one will also apply.

This definition is unproblematic as long as we remain in the realm of abstract objects and do not try to relate the formal systems of our analysis to the real world. However, empirical import is a crucial aspect of both principles discussed here. Claims about the equivalence between HP and the FEP are not merely mathematical but also concern mutual applicability with regard to real-world systems. This kind of empirical equivalence can be summarized by the statements that a free energy principle for biological systems ‘is just a delicate reconstruction of the principle of least action’ [21] and that ‘living systems conform to Hamilton’s principle of least action via active inference’ [2] (Box 2).

We can make sense of the equivalence claim in light of the FEP’s empirical commitment by considering the scope of both principles. In the existing literature on the FEP [55], the scope of a model is commonly understood as the set of phenomena that it is intended to capture (though this usage is closer to what philosophers of science [56,57] label as a generality—’a measure of how many phenomena a model or set of models successfully relate to’ rather than the ‘aspects of the target the model is intended to capture’ [57] (p. 180)). Taking this into consideration, we can propose that the two principles are equivalent if, and only if, their scopes overlap in such a way that whenever one of the two principles is applicable to some system, so will the other. Effectively, this definition is itself logically equivalent to the claim that the FEP and HP are equivalent if, and only if, their scopes are identical. It is this claim that we label as a strong reading of the relationship between the two principles.

#### Dilemma for the Strong Reading

We believe that adopting the strong reading of equivalence between the principles commits any FEP advocate to endorsing a set of mutually incompatible propositions:

1. The scope of application of the FEP includes all biological systems;
2. The scope of application of HP does not include biological systems;
3. The scopes of application of theoretical constructs are equivalent *iff* the theoretical constructs are logically equivalent;
4. The FEP and HP are logically equivalent.

As we can see, proposition (4) cannot be true if propositions (1) and (2) are also both simultaneously true. This leads to a dilemma for any proponents of the strong reading. The first horn of this dilemma is that, if the FEP is logically equivalent to HP (proposition 4 is true), then it does not apply to real (non-conservative) biological systems (so proposition 1 is false). The second horn is that, if the FEP does apply to biological systems (proposition 1 is true), then it cannot be logically equivalent to HP (proposition 4 must be false). We believe that this dilemma conclusively shows that the strong reading should not be adopted or charitably ascribed to the proponents of the FEP.

To show that this is the case, we will need to focus only on propositions (1) and (4) since, as was established in Section 2, we believe that there are good, independent reasons to take proposition (2) as being true. Although this is sometimes contested in the FEP literature, where assertions such as ‘the FEP […] can be applied to any biological system in general (much like Hamilton’s principle of least action)’ [28] (p. 190) can be found, a coherent argument or proof demonstrating the truth of these statements is never presented. We have already addressed these claims in Section 3.2, so we will not reiterate them here. It is enough to say that a problem arises if HP is used for modeling biological processes. When the motion of a system is based on symmetric differential equations and there is no dissipation of energy, HP can be straightforwardly applied. However, if a system is dissipative (does not conserve energy), then the equations of motion are not symmetric, and special modifications beyond HP are required to circumvent this fact. In our eyes, this is enough to secure proposition (2).

Let us now turn to proposition (1) which concerns the FEP’s scope. To investigate the truth of proposition (1) we need to identify the range of biological systems and phenomena compliant with the FEP. However, the FEP literature is very inconsistent about the range of phenomena that the principle is applicable to. For example, the FEP is routinely applied to social as well as cultural phenomena [58], thus obfuscating what sort of characteristics are key for distinguishing biological systems from non-biological ones and creating confusion about the range of characteristics that biological systems are assumed to exhibit according to the FEP. As far as the complexity of the modeled systems is concerned, both variational formulas can be applied to any system with an arbitrary number of degrees of freedom. It is crucial, though, that the system’s underlying dynamics are conservative. This assumption is made explicit in the case of modeling biological complexity using Hamiltonian dynamics [59] (pp. 44–45). Similarly, in FEP modeling, the modeled *non-biological* phenomena are either explicitly (conservation of cultural tropes, see [60]) or implicitly (maximization of adaptive fitness, see [61]) assumed to be conservative. These modeling idealizations or simplifications render the FEP simulations HP-compliant but create confusion about the (non-)conservatory nature of the modeled phenomena. Finally, as we have already covered in Section 3.2, some publications explicitly acknowledge that the underlying phenomena are non-conservative and thus need to be modeled as open and dissipative [43]. However, they are modeled as dissipative in a simple linear manner, which is not a valid approximation for a wide class of non-conservative, non-linearly dissipative phenomena, which arguably include the processes of life.

We can cut through the confusion by focusing on one simple observation, namely that if the FEP was truly a general variational principle for living things (understood as time-irreversible systems), then it would be the first of its kind. Proponents of the principle could argue that, although it does not fulfill the role of a general principle, the FEP can secure the status of a *special* principle without reinventing thermodynamics. After all, the modeling literature is ripe with examples of ad hoc modeling assumptions and idealizations [8], used to construct various special variational principles for non-equilibrium thermodynamics. Furthermore, there arguably exist many cases of dissipative systems for which the action functional can be found [7]. Thus, the claim that the FEP successfully manages—for the first time in history—to specify the action functional for a special subset of biological systems is not as outlandish as it may seem at first.

However, even this ameliorated claim presents problems for the FEP. Firstly, it would effectively mean that it is at most *partially* (i.e., only materially) equivalent to HP, i.e., that the scopes of the two principles are not identical but consist of only partially overlapping sets of systems. While even this limited claim would still be a moderate success for the FEP and its defenders, it also means that they would carry the burden of proof for demonstrating and delineating the overlap between the sets of systems for which the two principles are applicable. Secondly, establishing even a partial overlap between those two sets could still prove a dead end for the proponents of the FEP. On one hand, if the overlap turned out to be relatively small, it would no longer be clear what can be gained from comparisons between the two principles. On the other hand, if the overlap between the scopes of the two principles turned out to be too large, then the equivalence between the FEP and HP would be strengthened at the cost of the FEP’s applicability to biological systems. The latter problem has been recently brought to light by Aguilera et al. [38], who show that the overlap between the two principles is substantial since both of them hold only for fully symmetric interactions and break down as soon as asymmetries arise. But these findings should not be celebrated in favor of the strong reading, as they really bring the dilemma facing proponents of the FEP into full light: Either the FEP is logically equivalent to HP, in which case the set systems it is applicable to does not include biological systems, or it is only partially (materially) equivalent to HP, in which case it is not a general principle and will be applicable only to a limited subset of living systems. Either result undermines the truth of proposition (1) and brings the postulate of logical (i.e., non-contingent) equivalence between the FEP and HP into question.

We will close this section by considering what happens when an FEP model is explicitly aimed at systems outside of HP’s scope. As we have mentioned in Section 3.2, existing attempts at accounting for morphogenesis explicitly assume that the systems of interest to the FEP are open and dissipative. This extends the FEP formalism *beyond* the scope of HP, effectively placing the two principles even further apart from each other. As we have previously established, one may choose to model dissipative systems in a systematic way but only if some well-behaved function describing this process can be specified or if the target phenomena turn out to not be dissipative after all. In order to keep the core FEP proposition in place—i.e., that all biological systems evolve towards minimum surprise understood as a path integral of action—we have to either assume that the loss of energy proceeds in a regular law-like fashion or that the ‘loss’ of energy occurs only between parts of a system that nonetheless conserves the energy on the whole.

This scenario exemplifies the more general kind of formal sleight of hand used by the proponents of the FEP to circumvent HP’s in-applicability to non-conservative systems. In general, modelers would prefer to deal exclusively with Lagrangian systems, i.e., systems for which all the relevant physical information about motion is available [7]. All conservative systems have a corresponding Lagrangian. Thus, a common modeling idealization is to carry on as if the Lagrangian can be specified. There are two possible ways to regard a non-Lagrangian (dissipative) system as being Lagrangian: Either the newly described system is an ‘approximation’ of a genuine Lagrangian system that is modeled with the addition of Rayleigh dissipation function (which itself cannot be derived from the Lagrangian or HP) or as a dissipative subsystem of a larger system that is genuinely Lagrangian [7]. In other words, the way to sidestep the inapplicability of HP to dissipative systems is either by assuming that we can easily specify the loss of energy (e.g., for Rayleigh dissipation function it will be proportional to the motion of the system) or by considering a dissipative system and its environment as a whole—that is, a larger system where the total energy cannot be lost [62].

The former option may be achieved, for instance, by decomposing a system’s dynamics into a dissipative (curl-free) component and a conservative (divergence-free) flow [38]. The first component describes a flow that is solenoidal (with no sources or sinks) and non-divergent (instantly forgetting its past). These characteristics fall under the scope of HP and can be used to describe symmetrical and itinerant dynamics. The second component, formalized as a gradient ascent of log-probability (negative surprise), is intended to cover these forces that allow a weakly mixing open system to resist dispersion and reach its non-equilibrium-steady-state. This flow depends on the amplitude of random fluctuations and the non-equilibrium-steady-state density expressed in terms of surprisal. The flow’s non-conservative property can be associated with the dissipation of heat incurred in the maintenance of the steady state [37]. The FEP’s innovation could lie in providing an approximation of the dissipative component for a certain class of mathematical objects, which are subject to random fluctuations but tend to converge onto their random global attractor. However, this interpretation would reinforce FEP’s distinctness from HP and in turn provoke questions regarding its empirical relevance for biological phenomena.

The latter option for including dissipative systems is pursued by Kuchling et al. [43], who take into consideration ensembles instead of individual particles. Switching from single particles to their ensembles is intended to average over dissipative flows as a part of a conservative super-system and treat them as inconsequential for the dynamics of the whole, which is thought to slowly but surely reach the least action state. However, when applied in a biological setting, this move means effectively black-boxing any non-linear interactions that may be at play in a large biological system and forcing a view that biological systems are in fact conservative super-systems composed of dissipative parts.

In conclusion, if HP and the FEP have an identical scope (reversible processes), establishing the latter’s relevance for a different kind of (time-irreversible) process requires an independent argument. This is because HP is applicable to all reversible, isolated, and conservative systems, but it is not applicable to non-equilibrium thermodynamic systems, hence it excludes biological systems. The FEP, on the other hand, is aimed at describing systems that are dissipative, open, and far from equilibrium. Therefore, the two principles are not logically equivalent.

### 4.2. Weak Reading

So far, we have shown that the strong reading of the relation between the FEP and HP in terms of equivalence results in a dilemma that no proponent of the FEP should be willing to accept. However, we believe that there is a more charitable and tenable reading of the supposed connection between the two principles, one which avoids the dilemma, finds theoretical support in recent literature, and promises to better capture the motivation behind postulating such a relationship in the first place. This weak reading, as we call it, appeals to *analogy* rather than equivalence.

Analogical relations have a long tradition within scientific practice as well as the philosophy of science [63]. The billiard ball model of a gas, in which the movements and collisions of gas molecules are presented as analogous to those of balls on a table, is perhaps the most famous example of a scientific analogy [64]. The concept of an analogy is quite intuitive since, as Frigg and Hartmann explain: ‘[a]t the most basic level, two things are analogous if there are certain relevant similarities between them’ [65]. The issue, of course, is specifying what the ‘relevant similarities’ are. In the philosophical literature, this is usually unpacked in terms of different similarity or resemblance relations obtained between analogous relata: systems, phenomena, models, etc. While there are many ways of unpacking the kind of resemblance involved [66], we can broadly categorize analogies as being based on the sameness or similarity of monadic properties (e.g., whether our system of interest and our model animal are both mammals) and of relations between parts of the analog systems (e.g., whether the distance between any two points on a map will correspond to a scaled up distance between any two points in the represented environment). Mary Hesse [67] has labeled such relations as *material analogies* (also referred to as *qualitative* analogies [68]) since they are defined or specified in terms of some concrete (i.e., non-abstract) and observable properties or features of the relevant physical systems. However, science is equally likely to appeal to formal models, which abstract the features of the targets away while aiming to preserve their structure in a pattern of abstract relationships. For Hesse, such models rely on *formal analogy*, such that two domains, systems, or formal models will be related by a formal analogy provided that they are ‘interpretations of the same formal theory’ [67] (p. 68). Here, ‘theory’ should be understood rather broadly, as can be seen in the example between Poiseuille’s and Ohm’s laws. Frig and Hartmann [65] present Hesse’s view of formal analogy as a relation obtaining between items that ‘are both interpretations of the same formal calculus’, which might be a better formulation of the view. The analogy between the flow of electric current in a wire, described by Ohm’s law, and the flow of an (ideal) fluid in a pipe, described by Poiseuille’s law is a common example because both are expressed by equations that are interpretations of the same mathematical formula: Δ*x = yz* (i.e., both equations have the same syntactic structure, but differ in terms of what their variables denote). Moving into the domain of cognitive science, the relationship between Hopfield’s artificial neural networks [69] and the Sherrington–Kirkpatrick spin glass model [70] is also one of formal analogy since both kinds of systems share the same formal structure and can be described using the same mathematical equation (for more on this, see [71]).

Before we move on, it is worth noting that the examples used above trade in *positive* analogies, i.e., ones defined in terms of properties or relations shared by both systems. However, Hesse (following [72]) also notes that it is possible to describe *negative* analogies that are defined in terms of properties or features that are not shared by the relata. A third type of *neutral* analogies describes relations among properties that are either not known to be tokens of either of the previous relations and remain open to investigation or are deemed irrelevant in a given context. Comparisons between analogous systems are often thought to have epistemic relevance for scientific reasoning, although the kind of epistemic support or role that positive analogies can play in scientific reasoning is an ongoing debate and an issue that we will return to later.

With these preliminaries in place, we can focus on the claim that the FEP and HP are, in some way, *formally analogous* to one another. Given what has been said so far, this claim should not raise too much controversy. After all, both principles are abstract, formal constructs spelled out in terms of a common variational approach and, as we have shown in Section 3, the problems they aim to solve are defined in terms of finding the optimal trajectory through some abstract space. The idea that HP and the FEP can be seen as interpretations of the same formal calculus is also supported by the arguments presented by Aguilera and colleagues [38], which we have discussed in the previous section. Nevertheless, although formal analogy is an attractive way to view the relationship between the two principles, there is an important question about the type of analogy (or indeed analogies)—positive, negative, or neutral—that holds between the two constructs. In order to illustrate the kind of upshot that the weak reading can bring to debates about the FEP and HP, we will now look closer at the most prominent claims about the analogies between the two principles.

#### Assessing the Analogies between the Free Energy Principle and Hamilton’s Principle

Recent literature on the FEP embraces the analogical relationship between the FEP and HP. Parr and colleagues go so far as to explicitly talk about ‘drawing analogies between Hamiltonian physics and Active Inference’, i.e., the framework built around the FEP [27] (p. 54). They go on to specify three distinct levels at which FEP and HP are similar.

Firstly, there is a supposed analogy between the way in which formulation of neural processing and behavior in terms of the FEP allows advanced mind sciences, just like the Lagrangian and Hamiltonian formulations of mechanics allowed for advancing physics. Secondly, the authors postulate that there is a ‘a more direct association between a Hamiltonian and probability measures’ [27] (p. 55) in terms of an analogy between the formal expressions of measures used in variational inference and in statistical mechanics, such as energy, which take the form of negative logarithms of probability. As Parr and colleagues explain, this opens the way for interpreting the notion of energy as ‘a measure of the improbability of any given configuration of a system.’ (p. 55). 

The third analogy between the free energy framework and Hamiltonian mechanics is their shared reliance on variational calculus. In a vindication of Section 2 and Section 3, the authors state that this connection ‘is most apparent when Hamiltonian physics is expressed as a principle of least Action, where Action refers to the integral of a Lagrangian over a path.’ [27] (p. 55). As they go on to explain, in this context, action is formalized as ‘a function of time whose output is the position and velocity of a particle on that path at that time. The path followed by a (deterministic) particle minimizes this Action.’ [27] (p. 49, footnote 3). This is likened to the dynamics of probabilistic inference, in which beliefs (understood as functions between hidden and observable states) are optimized by minimizing the function of free energy. In Parr et al.’s own words: ‘this places both [FEP and HP] in the context of variational calculus.’ [27] (p. 55). 

These analogies are not of a piece. Not only are they not cast on the same level of analysis, but they also differ in terms of the roles that the analogical relation is supposed to play in scientific reasoning. Although all the relations invoked by Parr and colleagues appear to be relations of positive similarities, only the latter two of them fit the notion of formal analogy, as described in the previous section. The second relation points to a formal analogy between the key variable used in statistical mechanics and active inference, both of which are expressed in terms of the same abstract quantity (negative log probability). Meanwhile, the third analogy focuses on the structure of the operations that are carried on this variable, i.e., defining it as a function that needs to be minimized. While these two relations may not offer the kind of exact analogy, in which the syntactic structure of the equations remains identical and only the names of the variables change between different applications (as in the case of Ohm’s and Poiseuille’s laws), they do nevertheless offer a less exact (or perhaps more coarse-grained) analogy in terms of the use of variational calculus, which is enough for a weak reading of the relationship between the FEP and HP. 

In short, the second and third of Parr et al.’s analogies are based on the fact that both the FEP and HP represent the same *type* of formal equation. Indeed, these formal analogies illustrate precisely why we label this relationship as *weak*; the relationship between the two principles pertains *only* to the similarity of their abstract mathematical structures and not to the scope of their intended applicability (as would be the case if the strong reading discussed earlier). This has implications for the kind of scientific reasoning that these analogies can support. Formal analogies are restricted to purely abstract inferences that can guide the development of the formal aspect of the FEP framework via the similarities to equations in statistical mechanics. However, they do not support inferences about the applicability of the principle or formally derived models to real-world phenomena.

What about the first of the analogies described by Parr and colleagues? Unlike the other two relations, this one is not a relationship based on the formal or syntactic properties of its relata but on the role that the two principles are supposed to play in their respective fields. The authors clarify what they are after by stating that: ‘the advance offered by Active Inference to the behavioral and life sciences is comparable to the advanced Lagrangian and Hamiltonian formulations offered to Newton’s accounts of mechanics’ [27] (p. 55). Hence, while the second and third of Parr et al.’s analogies were concerned with the significance of these relations for the sake of theory development (e.g., establishing a formal analogy with HP to facilitate the development of formulations derived from FEP), this analogy is concerned with the significance of FEP (and the wider formal framework it is embedded in) for the disciplines investigating the brain and the mind. In other words, the second and third analogies concern the intra-theoretical status of the two principles within their respective theories, but the first analogy is concerned with the principles’ epistemic role for the theorists themselves. Crucially, nothing about the analogies concerning the theoretical status of the principles warrants any kind of inference about their epistemic role within their respective fields. Whether or not FEP will prove to be transformative for the disciplines making up cognitive science does not depend on and cannot be inferred from its formal analogies with HP.

The epistemic role of a hypothesis is determined, among other things, by how applicable it is in scientific reasoning and how much it enhances the scientific understanding of some phenomenon in a given domain. This is something that not only cannot be known *a priori* but also cannot be read off from the formal properties of the equations alone (though researchers can, of course, anticipate this based on their domain knowledge). We can illustrate this by returning to the example of the Sherrington–Kirkpatrick spin-glass model’s application within connectionism. While the invention of Hopfield networks was one of the turning points in research on artificial neural networks, it was nearly impossible to predict the developments that would follow (e.g., their use in simulating memory recall or opening the road towards the invention of unrestricted and restricted Boltzmann machines) based purely on the formal analogy between the two models. Whether or not formally analogous models will play the same epistemic role and enjoy the same epistemic status within distant domains can only be evaluated *a posteriori*. Importantly, such evaluations tend to depend on a model’s applicability in a particular domain (as in the case of the success of the Hopfield model) and not its formal similarities to constructs in other domains of knowledge. This is yet another reason why the analogical interpretation of the relationship between HP and FEP is weaker than the strong interpretation in terms of equivalence.

## 5. Conclusions

Our aim in this paper was to investigate the nature of the often-cited relationship between the FEP and the HP. As we have argued, the two principles are not equivalent to each other, as they are not applicable to the same set of systems. Instead, we have defended a *weaker* interpretation of the relationship between these constructs, one spelled out in terms of a formal analogy. More precisely, we propose that the FEP and HP are *not strictly* analogous to each other because they are not interpretations of the same token formal equation. Rather, following the claims made by Parr et al. [27], we propose that *HP and FEP are tokens of the same type of variational formula* that aims to minimize an energy variable expressed as a negative logarithm of probability.

Although this *week reading* of the claims about the similarity between the FEP and HP does shed light on the nature of the relationship between the two principles, it does not answer all the questions raised in the subject literature. An important issue that we have barely touched upon is the epistemic role and status of the FEP within its wider framework as well as in cognitive sciences more generally. As we have pointed out in the previous section, this problem is largely orthogonal to the question of the formal similarity between the two principles. While space limitations prevent us from addressing this issue here, we do hope that the analysis and arguments we presented here will offer a useful steppingstone for any researchers interested in that question.

## References

[1] Friston K., Laumond J.-P., Mansard N., Lasserre J.-B. (2017). The Variational Principles of Action. Geometric and Numerical Foundations of Movements.

[2] Ramstead M.J.D., Badcock P.B., Friston K.J. (2018). Answering Schrödinger’s Question: A Free-Energy Formulation. Phys. Life Rev..

[3] Da Costa L., Friston K., Heins C., Pavliotis G.A. (2021). Bayesian Mechanics for Stationary Processes. arXiv.

[4] Fortier M., Friedman D., Friston K. (2018). Of Woodlice and Men: A Bayesian Account of Cognition, Life and Consciousness. An Interview with Karl Friston. ALIUS Bull..

[5] Kim C.S. (2021). Bayesian Mechanics of Perceptual Inference and Motor Control in the Brain. Biol. Cybern..

[6] Berdichevsky V. (2009). Variational Principles. Variational Principles of Continuum Mechanics.

[7] dell’Isola F., Placidi L., Pfeiffer F., Rammerstorfer F.G., Salençon J., Schrefler B., Serafini P., dell’Isola F., Gavrilyuk S. (2011). Variational Principles Are a Powerful Tool Also for Formulating Field Theories. Variational Models and Methods in Solid and Fluid Mechanics.

[8] Ván P., Kovács R. (2020). Variational Principles and Nonequilibrium Thermodynamics. Philos. Trans. R. Soc. Math. Phys. Eng. Sci..

[9] Helrich C. (2007). Is There a Basis for Teleology in Physics?. Zygon.

[10] Escamilla-Rivera C., Fabris J.C. (2021). The Possibility of a Non-Lagrangian Theory of Gravity. Universe.

[11] Rao J.S., Rao J.S. (2011). Energy Methods. History of Rotating Machinery Dynamics.

[12] Feynman R., The New Millen (2013). The Feynman Lectures on Physics. Volume II, Mainly Electromagnetism and Matter.

[13] Badur J., Badur J., Sieniutycz S., Farkas H. (2005). An Introduction to Variational Derivation of the Pseudomomentum Conservation in Thermohydrodynamics. Variational and Extremum Principles in Macroscopic Systems.

[14] Longo G., Montévil M., Kauffman S. (2012). No Entailing Laws, but Enablement in the Evolution of the Biosphere. Proceedings of the 14th Annual Conference Companion on Genetic and Evolutionary Computation, Philadelphia, PA, USA, 7–11 July 2012.

[15] Kondepudi D., Prigogine I. (2014). Modern Thermodynamics: From Heat Engines to Dissipative Structures.

[16] Dodin I.Y., Zhmoginov A.I., Ruiz D.E. (2017). Variational Principles for Dissipative (Sub)Systems, with Applications to the Theory of Linear Dispersion and Geometrical Optics. Phys. Lett. A.

[17] Lazo M.J., Krumreich C.E. (2014). The Action Principle for Dissipative Systems. J. Math. Phys..

[18] Friston K., FitzGerald T., Rigoli F., Schwartenbeck P., Pezzulo G. (2017). Active Inference: A Process Theory. Neural Comput..

[19] Friston K. (2008). Hierarchical Models in the Brain. PLoS Comput. Biol..

[20] Friston K. (2009). The Free-Energy Principle: A Rough Guide to the Brain?. Trends Cogn. Sci..

[21] Friston K. (2012). A Free Energy Principle for Biological Systems. Entropy.

[22] Friston K., Stephan K.E. (2007). Free-Energy and the Brain. Synthese.

[23] Friston K., Kiebel S. (2009). Predictive Coding under the Free-Energy Principle. Philos. Trans. R. Soc. Lond. B Biol. Sci..

[24] Friston K., Daunizeau J., Kilner J., Kiebel S.J. (2010). Action and Behavior: A Free-Energy Formulation. Biol. Cybern..

[25] Calvo P., Friston K. (2017). Predicting Green: Really Radical (Plant) Predictive Processing. J. R. Soc. Interface.

[26] Ramstead M., Kirchhoff M., Friston K. (2019). A Tale of Two Densities: Active Inference Is Enactive Inference. Adapt. Behav..

[27] Parr T., Pezzulo G., Friston K.J. (2022). Active Inference: The Free Energy Principle in Mind, Brain, and Behavior.

[28] Ramstead M.J.D., Constant A., Badcock P.B., Friston K.J. (2019). Variational Ecology and the Physics of Sentient Systems. Phys. Life Rev..

[29] Friston K. (2005). A Theory of Cortical Responses. Philos. Trans. R. Soc. Lond. B Biol. Sci..

[30] Mann S.F., Pain R., Kirchhoff M.D. (2022). Free Energy: A User’s Guide. Biol. Philos..

[31] Buckley C., Kim C.S., McGregor S., Seth A. (2017). The Free Energy Principle for Action and Perception: A Mathematical Review. J. Math. Psychol..

[32] Bruineberg J., Dolega K., Dewhurst J., Baltieri M. (2021). The Emperor’s New Markov Blankets. Behav. Brain Sci..

[33] Millidge B., Seth A., Buckley C.L. (2021). A Mathematical Walkthrough and Discussion of the Free Energy Principle. arXiv.

[34] Bogacz R. (2017). A Tutorial on the Free-Energy Framework for Modelling Perception and Learning. J. Math. Psychol..

[35] Friston K.J., Daunizeau J., Kiebel S.J. (2009). Reinforcement Learning or Active Inference?. PLoS ONE.

[36] Hesp C., Smith R., Parr T., Allen M., Friston K.J., Ramstead M.J.D. (2021). Deeply Felt Affect: The Emergence of Valence in Deep Active Inference. Neural Comput..

[37] Friston K. (2019). A Free Energy Principle for a Particular Physics. arXiv.

[38] Aguilera M., Millidge B., Tschantz A., Buckley C.L. (2022). How Particular Is the Physics of the Free Energy Principle?. Phys. Life Rev..

[39] Webb S., Goliński A., Zinkov R., Siddharth N., Rainforth T., Teh Y.W., Wood F. (2018). Faithful Inversion of Generative Models for Effective Amortized Inference. Advances in Neural Information Processing Systems 31 (NeurIPS 2018), Proceedings of the 32nd International Conference on Neural Information Processing Systems, Montréal, QC, Canada, 3–8 December 2018.

[40] Clark A. (2013). Whatever next? Predictive Brains, Situated Agents, and the Future of Cognitive Science. Behav. Brain Sci..

[41] Rao R., Ballard D. (1999). Predictive Coding in the Visual Cortex: A Functional Interpretation of Some Extra-Classical Receptive-Field Effects. Nat. Neurosci..

[42] Encyclopedia Britannica (2016). Morphogenesis.

[43] Kuchling F., Friston K., Georgiev G., Levin M. (2020). Morphogenesis as Bayesian Inference: A Variational Approach to Pattern Formation and Control in Complex Biological Systems. Phys. Life Rev..

[44] Cline D., Sarkis M. (2017). Variational Principles in Classical Mechanics.

[45] Friston K., Michael L., Biswa S., Giovanni P. (2015). Knowing One’s Place: A Free-Energy Approach to Pattern Regulation. J. R. Soc. Interface.

[46] Nesbet R.K. (2005). Variational Principles and Methods in Theoretical Physics and Chemistry.

[47] Strutt J.W. (1873). Some General Theorems Relating to Vibrations. Proc. Lond. Math. Soc..

[48] Galley C.R. (2013). Classical Mechanics of Nonconservative Systems. Phys. Rev. Lett..

[49] Georgiev G., Georgiev I. (2002). The Least Action and the Metric of an Organized System. Open Syst. Inf. Dyn..

[50] de Oliveira C., Werlang T. (2007). Ergodic Hypothesis in Classical Statistical Mechanics. Rev. Bras. Ensino Física.

[51] Lombardi O., Fortin S., López C., Holik F. (2019). Quantum Worlds: Perspectives on the Ontology of Quantum Mechanics.

[52] Nicol M., Petersen K., Meyers R.A. (2011). Ergodic Theory: Basic Examples and Constructions BT—Mathematics of Complexity and Dynamical Systems.

[53] Annila A. (2023). Chiral Conformity Emerges from the Least-Time Free Energy Consumption. Interface Focus.

[54] Mendelson E. (1987). Introduction to Mathematical Logic.

[55] Sims A. (2016). A Problem of Scope for the Free Energy Principle as a Theory of Cognition. Philos. Psychol..

[56] Levins R. (1966). The Strategy of Model Building in Poupation Biology. Conceptual Issues in Evolutionary Biology.

[57] Matthewson J., Weisberg M. (2009). The Structure of Tradeoffs in Model Building. Synthese.

[58] Badcock P.B., Ramstead M.J.D., Sheikhbahaee Z., Constant A. (2022). Applying the Free Energy Principle to Complex Adaptive Systems. Entropy.

[59] Sleeman B.D. (1989). Complexity in Biological Systems and Hamiltonian Dynamics. Proc. R. Soc. Lond. Math. Phys. Sci..

[60] Veissière S.P.L., Constant A., Ramstead M.J.D., Friston K.J., Kirmayer L.J. (2020). Thinking through Other Minds: A Variational Approach to Cognition and Culture. Behav. Brain Sci..

[61] Hesp C., Ramstead M., Constant A., Badcock P., Kirchhoff M., Friston K., Georgiev G.Y., Smart J.M., Flores Martinez C.L., Price M.E. (2019). A Multi-Scale View of the Emergent Complexity of Life: A Free-Energy Proposal. Evolution, Development and Complexity.

[62] Wang Q.A., Wang R. (2018). A True Least Action Principle for Damped Motion. J. Phys. Conf. Ser..

[63] Bartha P., Zalta E.N. (2022). Analogy and Analogical Reasoning. The Stanford Encyclopedia of Philosophy.

[64] Hesse M. (1953). Models in Physics. Br. J. Philos. Sci..

[65] Frigg R., Hartmann S., Zalta E.N. (2020). Models in Science. The Stanford Encyclopedia of Philosophy.

[66] Toon A. (2012). Similarity and Scientific Representation. Int. Stud. Philos. Sci..

[67] Hesse M. (1963). Models and Analogies in Science.

[68] Sacksteder W. (1974). The Logic of Analogy. Philos. Rhetor..

[69] Hopfield J.J. (1982). Neural Networks and Physical Systems with Emergent Collective Computational Abilities. Proc. Natl. Acad. Sci. USA.

[70] Sherrington D., Kirkpatrick S. (1975). Solvable Model of a Spin-Glass. Phys. Rev. Lett..

[71] Churchland P., Sejnowski T. (1992). The Computational Brain.

[72] Keynes J.M. (1921). A Treatise on Probability.

